# The use of semi-quantitative ultrasound elastosonography in combination with conventional ultrasonography and contrast-enhanced ultrasonography in the assessment of malignancy risk of thyroid nodules with indeterminate cytology

**DOI:** 10.1186/s13044-014-0009-8

**Published:** 2014-12-05

**Authors:** Massimo Giusti, Claudia Campomenosi, Stefano Gay, Barbara Massa, Enzo Silvestri, Eleonora Monti, Giovanni Turtulici

**Affiliations:** Endocrine Unit, IRCCS Azienda Ospedaliera Universitaria San Martino – IST Istituto Nazionale per la Ricerca sul Cancro, Genoa, Italy; Cytopathology and Pathology Unit, IRCCS Azienda Ospedaliera Universitaria San Martino – IST Istituto Nazionale per la Ricerca sul Cancro, Genoa, Italy; Radiology Unit, Ospedale Evangelico, Genoa, Italy; UO Clinica Endocrinologica, Viale Benedetto XV, 6, I-16100 Genoa, Italy

**Keywords:** Thyroid nodules, Indeterminate cytology, Ultrasosonography, Ultrasound elastosonography, Strain index, Contrast-enhanced ultrasonography, Cytological–histological correlation, ROC analysis

## Abstract

**Background:**

The pre-surgical selection of thyroid nodules with indeterminate cytology (Thy 3 according to British Thyroid Association) after fine-needle aspiration biopsy (FNAB) is currently required in order to reduce unnecessary total thyroidectomy. The objective of our study was to use a surgical series of Thy 3 nodules to evaluate the predictive role of ultrasound elastosonography (USE) and contrast-enhanced ultrasonography (CEUS) in pre-surgical diagnoses of malignancy.

**Subjects and methods:**

We enrolled 63 patients with Thy 3 nodules in which cytological–histological correlation was available. The ELX 2/1 strain index was obtained by means of semi-quantitative USE, which was performed before surgery in addition to conventional ultrasonography (US) and contrast-enhanced US (CEUS) on the Thy 3 nodules. The ELX 2/1 strain index, a five-item US score and both peak (P) index and time to peak (TTP) index from CEUS were correlated with the histological results. After surgical diagnosis, the data were analysed by using a receiver-operating characteristic (ROC) curve.

**Results:**

Histology was benign in 50 and malignant in 13 Thy 3 nodules. No difference in maximal diameter was noted between benign (22.8 ± 1.6 mm) and malignant (18.9 ± 2.9 mm) nodules. Significant correlations were found between histology and cumulative US findings (p=0.005), ELX 2/1 index (p=0.002), P index (p=0.01) and TTP index (p=0.02). On analysing data from US, USE and CEUS, significant ROC areas under the curve were observed (p<0.0001). A cut-off value was set for US (>2), ELX 2/1 (>0.95), P index (<0.99) and TTP index (>0.98) scores. The diagnostic power of the cumulative pre-surgical analysis of Thy 3 nodules with US, USE and CEUS, considering the experimental cut-off points obtained from the ROC curves was: sensitivity 64%, specificity 92%, PPV 75% and accuracy 84%.

**Conclusion:**

The ELX 2/1 index in conjunction with the US score can be useful in orienting surgical strategies in Thy 3 nodules. The information added by CEUS is less sensitive than that provided by US and USE. The use of a cut-off based on histology can reduce thyroidectomy. Observation should be the first choice when not all instrumental results are suspect.

## Introduction

The prevalence of diagnoses of thyroid nodules often varies according to the method of examination used [1-3]. The current thyroid nodule and thyroid cancer epidemic can be explained by the worldwide diffusion of ultrasonography (US) equipment [4,5]. When a nodule is found, the most important clinical problem is to exclude malignancy, which accounts for approximately 5%-15% of all thyroid nodules [6-8]. A combination of clinical factors (age, sex, exposure to radiation, familial traits) and US features determines whether or not the clinician should proceed with further tests or observation. The accuracy of US in predicting thyroid cancer has recently been reviewed by Brito et al. [9]. Only two US findings – spongiform and cystic thyroid nodule features – seem to provide sufficient probability to help rule out cancer and to suggest observation at first, while all other US findings, when assessed individually, might not be able to rule in or rule out malignancy, owing to their modest likelihood ratio [9]. Therefore, in order to distinguish malignant from benign thyroid nodules, cytological investigation by means of fine-needle aspiration biopsy (FNAB) must be performed under US guidance in nodules larger than 10 mm or with suspicious US findings [10-13]. Thyroid cytology is usually reported both descriptively and as suggested categories with different risks of malignancy. Both the Bethesda system and the British Thyroid Association (BTA) category (Thy 1 – Thy 5) are used. The ultimate aim of FNAB is to reassure the patient and to avoid surgery if not otherwise indicated [13]. FNAB yields useful cytological results in about 80% of cases, but has several weaknesses, including false negative (about 1-2%), non-diagnostic (3-16%) and indeterminate (follicular lesions; 6-20%) results [13-16]. The low risk of underestimating a thyroid cancer supports the recommendation for repeat thyroid nodule evaluation 2–4 years after initial benign (Thy 2) FNAB [16], while the very high probability (77-100%) [17-21] of cancer in nodules “suspicious for malignancy” (Thy 4) obliges surgery, as in the case of findings that are “diagnostic of malignancy” (Thy 5). Core-needle biopsy seems more useful than FNAB repetition in reducing non-diagnostic (Thy 1) cytology [22], but its utility in cases of indeterminate cytology after FNAB is still debated [22,23]. As indeterminate lesions (Thy 3) are associated with an approximately 25% risk of malignancy [12,17,19,24-27], histological examination is still required by the current guidelines of thyroid societies. Rago et al. [27] recently reported an overall good prognosis in Thy 3 lesions with malignant histology, which suggests the possibility that more exhaustive pre-surgical evaluation might reduce unnecessary (about 70% of cases) surgery. In this context, the role of US in distinguishing malignant from benign Thy 3 nodules is still uncertain, though in the large series of Thy 3 nodules examined in Rago’s study, blurred nodule margins and spots of microcalcification were significantly associated with malignancy.

The introduction of novel diagnostic tools may provide a more reliable approach to assessing the risk of malignancy in Thy 3 nodules. Recently, the determination of somatic mutation in FNAB specimens from Thy 3 nodules has been proposed [17,28,29], and may also help in deciding the extent of surgery [30]. However, the role of molecular screening could be overestimated, as this technique increases FNAB sensitivity from 67% to only 75% in indeterminate lesions [31]. On the other hand, a search for several gene expressions conducted in 326 nodules with indeterminate cytology has demonstrated the substantial impact of this approach on clinical care recommendations, though site-to-site variation exists [32] and this evaluation is not yet universally available.

US elastography (USE) and contrast-enhanced US (CEUS) are another two innovative techniques under evaluation for the detection of malignancy in thyroid nodules, but they still need validation. USE has been likened to “electronic palpation” and provides reproducible stiffness measurements even in otherwise non-palpable thyroid lesions [33]. At present, heterogeneity among different USE technologies (difference in compression source, modality of processing, stiffness expression), equipment and applications explains why USE is not yet part of routine nodule management [19,33]. USE should primary be implemented in the pre-surgical differential diagnosis between benign and malignant nodules; however, its accuracy is debated and surgery has not always been taken as the reference in evaluating USE data [17-21,25]. In addition, a certain risk of false negative results has been reported, especially in cases of follicular [34,35] or medullary thyroid cancer [36]. CEUS is a dynamic evaluation which enables thyroid nodules to be characterized by studying vascular enhancement patterns, which it does better than conventional US. Some authors have reported that the absence of ring enhancement and the presence of heterogeneous enhancement [37-39] or a shorter time to peak of the perfusion curve [40] could characterize malignant nodules. However, these data are still controversial [19,41].

The pre-surgical diagnostic role of USE [17-19,21,25,42,43] and CEUS [19,37] in nodules with indeterminate cytology has been considered uncertain because of the low number of these nodules in the series analyzed. Two recently published studies have focused on USE in indeterminate nodules. In 169 patients in whom histology was available after qualitative USE, Rago et al. [27] reported that high elasticity is closely associated with benign histology, with a negative predictive value of 97%. In addition, in a series of 270 nodules with atypia of indeterminate significance evaluated by means of semi-quantitative USE, Cakir et al. [44] reported a higher median strain index in the malignant group than in the benign group. To our knowledge, no data are available in the literature on large series of Thy 3 nodules evaluated by CEUS in order to judge the pre-surgical role of this technique.

The aim of the present study was to retrospectively evaluate the pre-surgical role of both USE and CEUS together with US findings in our series of patients with indeterminate cytology and known histology. The construction of a validate cut-off for numerical indices could be used to avoid surgical procedures in Thy 3 patients in whom all tests (US, USE, CEUS) yield “benign” results. The cost and time consumption of these techniques could be lower than those of unnecessary thyroid surgery.

## Materials and methods

### Patients

This prospective study enrolled 78 consecutive patients (60 female, 18 male; age: 20–82 yrs; mean ± SD: 55.4 ± 14.7 yrs) with thyroid nodules observed at the out-patient Thyroid Cancer Unit. On FNAB, performed as previously reported [19], all patients had an indeterminate thyroid lesion (Thy 3) according to the 2009 BTA classification. These patients were considered candidates for surgery in accordance with the cytological result. In 47% of these patients, the Thy 3 nodule was found in a multinodular goitre, while 53% had a uninodular goiter. In 8 patients, laboratory data were compatible with Hashimoto’s thyroiditis, while one patient had a single Thy 3 nodule in a diffuse toxic goiter. Levothyroxine was being taken by 20% of patients, either for hypothyroidism (n=3) or as a TSH-reducing therapy (n=13). Three patients were on methimazole therapy for pre-toxic goiter. All patients underwent laboratory evaluations. TSH and free-T4 (f-T4) were measured by means of ultra-sensitive chemiluminescence immunoassay (Roche Diagnostics, Mannheim, Germany). Normal ranges are: 0.3-4.2 mIU/l for TSH and 12.0-22.0 pmol/l for f-T4. Thyroperoxidase antibodies (TPOAb) were evaluated by means of the Dia Sorin assay (Saluggia, Italy); concentrations <100 mIU/l were regarded as negative. Serum calcitonin (CT) was assayed by chemiluminescence immunoassay (Dia Sorin); in our laboratory, the upper limit of the normal CT range is 10 ng/l. All patients were invited to undergo further sonographic evaluation. All patients gave their informed consent to participate in the study. The collection of patient’s data and subsequent analysis was performed in compliance with the Helsinki declaration and was approved by University of Genoa Ethical Committee.

### Thyroid US, USE and CEUS

All patients were examined in the supine position with the neck extended. Scans of both thyroid lobes and isthmus were obtained in both transverse and longitudinal planes. The Thy 3 nodules were examined by means of conventional high-resolution US with a colour-Doppler module (MyLab40, Esaote Biomedica, Genoa) equipped with a 7.5 MHz linear probe. In 23 subjects, a further FNAB was performed with the aid of this equipment. As previously reported [19] and in accordance with US guidelines [45], the following parameters were investigated: echogenicity *vs.* non-nodular tissue, presence or absence of halo sign, presence or absence of microcalcifications, and flow pattern of the nodule. All USE examinations were performed by the same operator (GT) by means of a MyLab 70 XvG US scanner (Esaote Biomedica) equipped with an LA-522 linear probe working in the range of 7–12 MHz and software for the quantification of the USE features of the tissue. Static and moving images were recorded, as already reported by Lyshchik et al. [34], at least 3 times in order to obtain mean values. The elasticity score (ELX 2/1) index was calculated at the same depth as the ratio between the elasticity feature of the selected region-of-interest (ROI) located on US-normal thyroid tissue and the ROI of the nodule under investigation. As previously reported [19], we considered the ELX 2/1 index directly reported on the screen of the equipment, as this is a less operator-dependent variable than the elasticity colour-scale extrapolated from breast tissue to the thyroid gland by some authors [18,25,35,46]. CEUS images were acquired by the MyLab 70 US scanner, as previously reported [19], by using a non-destructive US mode after bolus injection of SonoVue (4.8 ml; Bracco, Milan). CEUS video-clips were digitally recorded and analysed by means of Q-Contrast software V.4.0. (Bracco). Time-intensity curves within selected ROI and colour maps were acquired. Nodule and healthy thyroid tissue values of peak contrast enhancement (Peak) and time to peak (TTP) were calculated. Peak and TTP are reported as indexes (Peak index, TTP index) derived from the ratio between the values from the ROI of the nodule and the ROI of normal thyroid tissue [19].

### Statistical analysis

Non-parametric tests were used to compare averages; the correlation coefficient r was calculated by means of Spearman correlation (Sr) (GraphPad 6.0 Software, San Diego, CA, USA). Data are reported as mean ± standard error of mean (SEM) if not otherwise reported. Significance was set at p ≤ 0.05. A US score (from 0 to 5) was arbitrarily calculated for the nodule under evaluation, with one point being assigned for the presence of each of the following radiological findings: solid, hypo-echoic, microcalcification, internal vascularisation, and irregular shape [19]. All cytological and histology diagnoses were made by a pathologist (BM) with 10 years’ experience in the pathologic analysis of thyroid cancer. After surgery, Thy 3 lesions were classified as malignant or benign. The diagnostic value of the ELX 2/1 index from USE or the P index and TTP index from CEUS in distinguishing between benign and malignant nodules was analysed by means of the receiver-operating characteristic (ROC) curve and calculated area under the curve. After using this curve to establish a cut-off point, we established sensitivity and specificity values and likelihood ratios. The cumulative results from US, USE and CEUS were evaluated for sensitivity, specificity, positive predictive value and accuracy. Thy 3 nodules that fitted all experimental cut-off points obtained from ROC curves were considered true positive if they proved malignant and false positive if they proved benign on histological examination. In addition Thy 3 nodules that did not fit all experimental cut-off points obtained from ROC curves were considered true negative if they proved benign and false negative if they proved malignant on histology.

## Results

At the time of FNAB evaluations, all patients had normal f-T4 (15.1 ± 0.3 pmol/l) and TSH (2.0 ± 0.2 mIU/l) levels. CT was in the normal range in all but two patients; in these two patients, CT levels above the upper limit of the normal range (20 ng/l and 27 ng/l) were recorded. Surgery was performed in 63 of the 78 patients (81%). Surgery was not performed in 19% of Thy 3 nodules for the following reasons: down-grading of BTA classification from Thy 3 to Thy 2 (benign lesion) after the second FNAB (n=6), patient lost (n=4), patient request for further follow-up (n=2), patient refusal (n=2) and severe comorbidity (n=1). After surgery, a benign histological diagnosis of the cytological Thy 3 nodule was found in 50 cases (25 hyperplastic nodules; 18 follicular adenomas; 6 Hürthle cell adenomas; 1 intra-thyroid parathyroid adenoma). In 9 of these 50 cases (18%) an extra-nodular focal lymphocytic thyroiditis pattern was found, and in 6 (12%) a micro-papillary carcinoma of the isthmus (n=1) or in the contralateral lobe (n=5) was incidentally found. In one patient, C-cell hyperplasia was described. A final diagnosis of malignancy in the cytological Thy 3 nodules was reported in 13 cases (21%) (5 papillary thyroid carcinomas; 5 follicular variants of papillary thyroid carcinomas; 2 follicular carcinomas; 1 medullary thyroid carcinoma). Table 1 reports the clinical details of patients with thyroid malignancy. No difference in maximal diameter was noted between Thy 3 nodules with benign (22.8 ± 1.6 mm) or malignant (18.9 ± 2.9 mm) histology.

**Table 1:** **Clinical and instrumental data on Thy 3 nodules with proven malignancy on histology**

**#**	**Age (yrs)**	**Sex**	**Nodule size (mm)**	**US (score)**	**USE (ELX 2/1)**	**CEUS P index TTP index**		**Histology (°)**	Tumour stage (*)
1	23	m	25	3	1.10	0.75	0.90	FTC	1
2	34	m	9	3	1.90	0.86	1.10	MTC	1
3	36	f	20	3	1.53	0.69	1.07	FvPTC	1
4	40	f	15	1	1.50	1.00	1.00	FTC	1
5	45	f	30	4	3.00	0.69	1.25	FvPTC	3
6	45	f	44	4	ne	0.82	1.62	FvPTC	3
7	46	f	7	4	1.00	0.60	0.63	PTC	1
8	46	f	9	3	1.30	0.95	1.20	FvPTC	1
9	50	m	13	3	1.80	0.33	1.00	PTC	3
10	54	m	20	3	1.70	0.90	2.40	PTC	1
11	66	m	23	5	1.50	0.80	1.01	PTC	1
12	71	f	7	3	1.90	0.77	1.00	PTC	1
13	74	f	22	3	2.00	0.90	1.00	PTC	1

(°) FTC = follicular thyroid carcinoma; MTC = medullary thyroid carcinoma; FvPTC = follicular variant of papillary thyroid carcinoma; PTC = papillary thyroid carcinoma.

(*) Tumour stage on diagnosis according to AJCC/UICC 2010 Seventh Edition criteria.

ne = not evaluable owing to coarse calcification (case #6).

**Figure 1:**
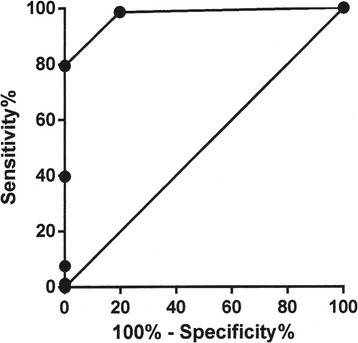
**ROC curve created with histology as a reference for distinguishing malignant from benign Thy 3 nodules according to US score.** The best cut-off was >2.

A significant correlation was seen between cumulative US findings and histology (n=63, Sr 0.35; p=0.005). A ROC curve was obtained (Figure 1) with an area under the curve (AUC) of 0.97 ± 0.01 (p<0.0001). By establishing a cut-off level that classified Thy 3 nodules with a US score greater than 2 as malignant, we were able to achieve a sensitivity of 79.0% and a specificity of 100%, with a 5.0 likelihood ratio.

**Figure 2:**
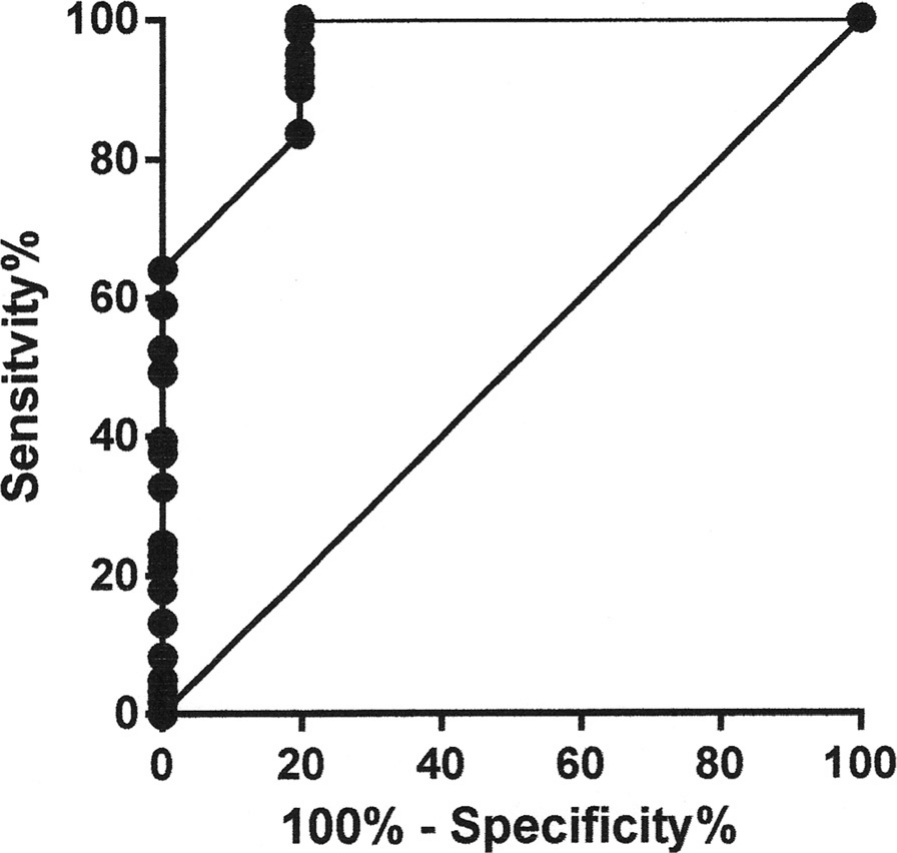
**ROC curve created with histology as a reference for distinguishing malignant from benign Thy 3 nodules according to USE ELX 2/1 strain index.** The best cut-off was >0.95.

USE was available in 61 Thy 3 nodules before surgery. In the remaining 2 cases, the ELX 2/1 index was unobtainable owing to coarse calcification of a 44 mm malignant nodule and the loss of data in a 23 mm benign nodule. A significant correlation was seen between the ELX 2/1 index and histology (Sr 0.39; p=0.002). A ROC curve was obtained (Figure 2) with an AUC of 0.95 ± 0.02 (p<0.0001). By establishing a cut-off level that classified Thy 3 nodules with an ELX 2/1 index greater than 0.95 as malignant, we were able to achieve a sensitivity of 83.6% and a specificity of 80.3%, with a 4.2 likelihood ratio. No significant correlation was found between the ELX 2/1 index and nodule (maximal) size or cumulative US findings (score).

**Figure 3:**
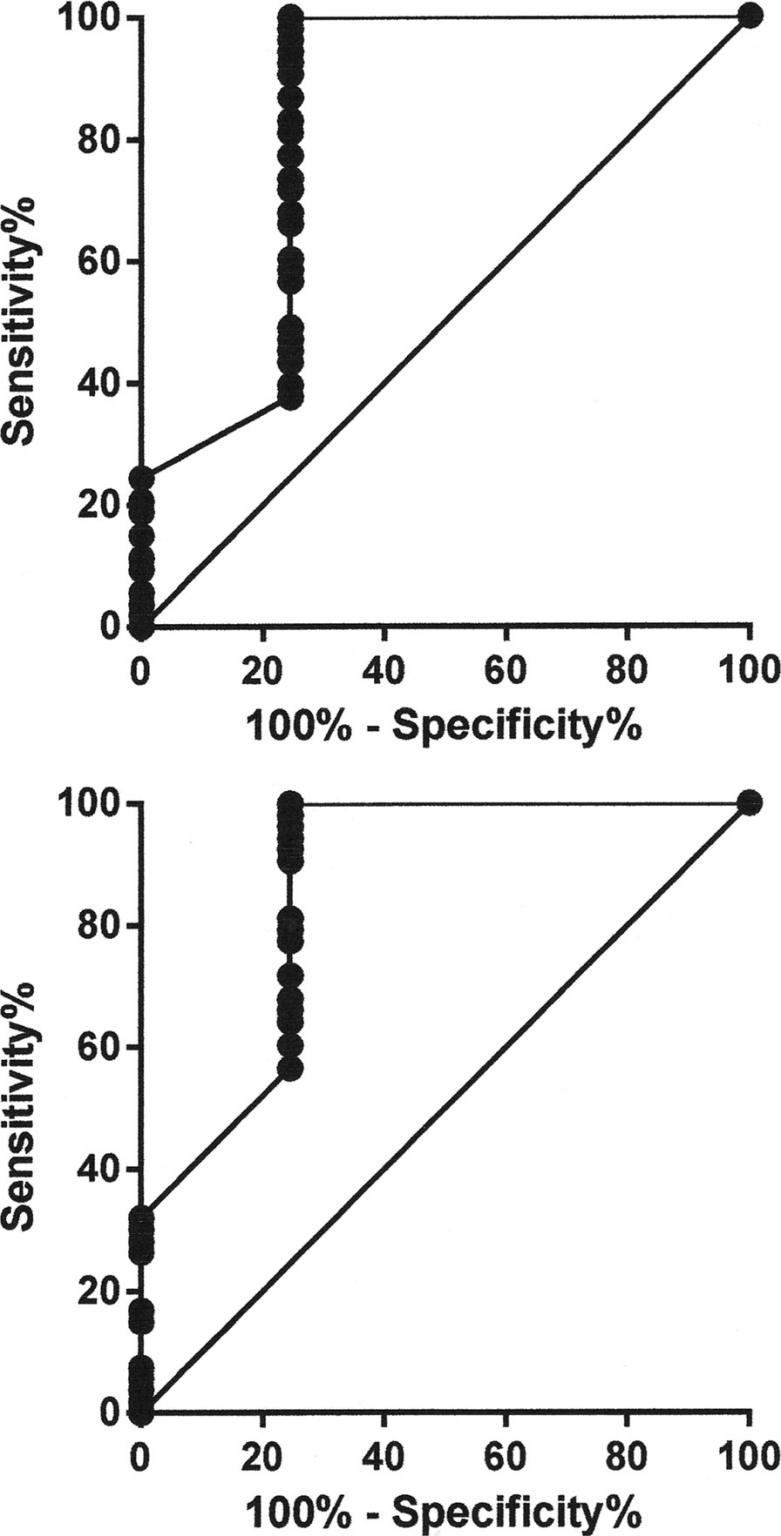
**ROC curve created with histology as a reference for distinguishing malignant from benign Thy 3 nodules according to CEUS.** The upper panel reports the P index; the best cut-off was <0.99. The lower panel reports the TTP index; the best cut-off was >0.98.

CEUS was performed on 53 Thy 3 nodules; no side effects were recorded during or immediately after injection of the contrast agent. CEUS could not be performed in 10 Thy 3 nodules owing to refusal of intravenous injection of the contrast agent (n=5), technical impossibility of recording adequate video-clips (n=3) and loss of data (n=2). All these Thy 3 nodules (size-range: 14–30 mm) had benign histology. A significant inverse correlation was observed between histology and P index (Sr −0.37; p=0.01) while a significant positive correlation was found between histology and TTP index (Sr 0.32; p=0.02). ROC curves were obtained for the P index and TTP index, with an AUC of 0.83 ± 0.04 (p<0.0001) and 0.86 ± 0.04 (p<0.0001), respectively (Figure 3). By establishing a cut-off level that classified Thy 3 nodules with a P index lower than 0.99 as malignant, we were able to achieve a sensitivity of 37.7% and a specificity of 75.5%, with a 1.5 likelihood ratio. By establishing a cut-off level that classified Thy 3 nodules with a TTP index greater than 0.98 as malignant, we were able to achieve a sensitivity of 56.6% and a specificity of 75.5%, with a 2.3 likelihood ratio. A significant inverse correlation was found between P index and US findings (score) (Sr −0.44; p=0.001). The peak index was not related to nodule size, while the TTP index was not related to either US findings (score) or nodule size.

A cohort of 51 Thy 3 cases underwent US-guided FNAB, USE and CEUS before surgery. In 9 out of 12 (75%) histologically malignant Thy 3 nodules, all indictors were positive for malignancy, while they were all positive in 5 out of 39 (13%) histologically benign Thy 3 nodules. The diagnostic power of the cumulative pre-surgical analysis of Thy 3 nodules by means of US, USE and CEUS, considering the experimental cut-off points obtained from ROC curves was: sensitivity 64%, specificity 92%, PPV 75%, and accuracy 84%.

## Discussion

The number of thyroid cancer diagnoses is currently increasing. This is probably the result of a reservoir of asymptomatic (subclinical) malignant nodular thyroid disease which is disclosed in parallel with the “epidemic” of thyroid nodules due to the increase in instrumental, mainly US, diagnostics. Current clinical efforts should be aimed at defining the nature (malignant or benign) of a given nodule and identifying the relatively rare malignant nodules, in order to reduce unnecessary invasive surgical procedures.

US, which is the most sensitive means of evaluating thyroid morphology, can evaluate the size and characteristics of non-palpable nodules, reveal lymph-node metastases and guide FNAB. It is well known that no single US pattern can be used alone as a definite criterion of malignancy [45], and individual US features are not now considered accurate predictors of thyroid cancer [9].

The present study demonstrates that, in Thy 3 nodules, nodule size is not a useful means of distinguishing malignant from benign nodules, as already reported by several other authors [12,27,47]. In a previous study involving five US parameters (hypoechogenic, solid, intra-nodular vascularization, microcalcifications, and irregular margins), we found that the diagnostic power of color-Doppler US was high; indeed, 100% of nodules in which four or five these parameters were positive proved to be malignant [19]. Similarly, on using the so-called “thyroid imaging reporting and data system”, Horvath et al. [48] found malignancy in 80% of nodules classified as probably malignant on US. A limitation of these studies, however, was the 5-14% of false negative results in nodules with a low number of suspicious US features [19,48]. In a very large series of Thy 3 nodules, Rago et al. [27] recently reported a significant association of three suspicious US features (blurred margins, spot microcalcifications and hypoechogeneicity) with malignancy. Moreover, in the present study, a significant correlation was found between cumulative US findings and histology, and the ROC curve analysis indicated that the presence of more than two suspicious US findings in Thy 3 nodules reached a sensitivity of 82% and a specificity of 100%, with a 5.0 likelihood ratio. In a series of 40 Thy 3 nodules with US available for review, Matthey-Gie et al. [49] reported that high nodule vascularity associated with ill-defined borders was a suspicious US finding linked to malignancy. In a series of 78 indeterminate lesions, Batawil and Alkordy [50] found that solid structure and irregular border were the most suspicious findings. Yoo et al. [26] examined a selected series of 249 nodules which met the criteria of atypia or follicular lesion of indeterminate significance and in which core-biopsy or surgery were used as references; they observed that taller-than-wide shape and marked hypoechogenicity were highly suspicious US findings. In 61 follicular neoplasms and 99 Hürthle cell neoplasms, Tutuncu et al. [51] did not report the role of cumulative US findings; however, they indicated that hypoechogenicity and microcalcification had the highest odds ratios. In sum, in Thy 3 nodules, US is helpful to the initial decision-making process, but combination with other evaluations is needed.

More recently, USE machines able to measure tissue hardness qualitatively (colour score), semi- quantitatively (strain ratio), or quantitatively (elasticity index, shear-wave velocity) have been introduced into the clinical setting in order to overcome the limitations of FNAB and US in thyroid nodules [33]. The efficacy of USE in distinguishing benign from malignant thyroid nodules varies widely among studies [17-20,25,34,35,46,47,52]). In a 2013 meta-analysis by Razavi et al. [53], in which 24 studies comprising 3531 nodules were evaluated and the results were compared with those yielded by US, USE was reported to be more sensitive (colour score 82%; strain ratio 89%) and specific (colour score and strain ratio 82%) than each individual US feature. However, in a previous study in which we evaluated a subset of 27 Thy 3 nodules with cytological-histological correlation by means of semi-quantitative USE, we were unable to distinguish these nodules from cytological benign (Thy 2) or malignant (Thy 5) nodules on the basis of the ELX 2/1 strain index [19]. The only reported finding was an increasing trend in low-range ELX 2/1 strain index values from Thy 2 to Thy 5 nodules [19]. In the present study, which involved a higher number of Thy 3 nodules, the ELX 2/1 strain index showed a significant correlation with histology, and the established cut-off level, which classified Thy 3 nodules with an ELX 2/1 index greater than 0.95 as malignant, showed better sensitivity of 83.6% and specificity of 80.3%, with a 4.2 likelihood ratio. In the study by Rago et al. [27], qualitative USE was performed in 169 Thy 3 nodules in which cytological-histological correlation was available, and low stiffness was found in nodules with benign diagnoses, the negative predictive value of 97% increasing the predictivity of US features. In several other studies on the pre-surgical utility of USE in thyroid nodules, the number of indeterminate lesions was limited, ranging from 9 to 58 [17,21,43,47,53], and separate data analysis was either not done [17] or, when done, was poorly understandable [21] or revealed low sensitivity (78%) and specificity (44%) [47]. Similarly, a very low specificity (6%) was observed in a study by Lippolis et al. [46], which evaluated 103 nodules with indeterminate cytology. On the other hand, in 270 nodules cytologically classified as atypia of undetermined significance by means of qualitative and semi-quantitative USE, Cakir et al. [44] reported favourable conclusions, suggesting the use of this technique in the pre-surgical evaluation of indeterminate nodules. The best strain index value in distinguishing between cytologically benign and cytologically malignant nodules reaches a sensitivity of 99% and a specificity of 96%.

Overall, the diagnostic utility of USE in Thy 3 nodules remains under evaluation. However, qualitative and semi-quantitative methods and definite cut-off values obtained in large numbers of subjects have yielded promising results (present study, [27,44]), thus supporting a less aggressive strategy, especially when no suspicious features are noted on US.

The latest technique involving US for the study of thyroid vascularization uses a microbubble contrast agent. It is well known that the thyroid gland has an abundant microvasculature, and that the parenchyma of normal thyroid shows rapid and uniform enhancement after intravenous injection of contrast agents. By contrast, the vascular structure of nodules differs from the normal pattern, and hence enhancement differs from that of the normal parenchyma. Argalia et al. [54] have shown that CEUS time-intensity curves can provide an indirect description of intra-nodular vascularization, which seems to be anarchic in malignant nodules. Literature data on the utility of CEUS in distinguishing malignant from benign nodules are controversial. Zhang et al. [37] reported that heterogeneous enhancement rendered a thyroid nodule suspicious for malignancy, while Friedrich-Rust et al. [41] reported that the time-intensity CEUS curve did not prove useful in distinguishing between benign and malignant nodules. Moreover, in our previous report [19], the P index and TTP index were found to be unrelated to cytological and histological results. More recently, two studies involving Chinese patients examined the role of CEUS in distinguishing between malignant and benign thyroid nodules. In 175 nodules, without histological reference in all cases, Deng J et al. [39] reported a CEUS sensitivity of 82% and a specificity of 85%. Their study was based on the impression that hypo-enhancement could be regarded as an indicator of malignancy. Ma et al. [38] studied the preoperative diagnostic role of CEUS combined with US in 172 nodules, all surgically removed. Both US and CEUS areas under the ROC curves were significant, but the best sensitivity (89%) and specificity (94%) was reached by considering five positive features (ring enhancement, homogeneity of enhancement, arrival time of the nodule at CEUS, microcalcifications, and halo sign on US) on combining US and CEUS.

To our knowledge, no studies on CEUS have focused on the subpopulation of indeterminate nodules. In our previous study on CEUS in thyroid nodules, CEUS was available in 17 indeterminate nodules with cytological-histological correlation [19]. Regarding the indicators P index and TTP index, no differences were noted among the nodules scored according to the Thy classification [19]. The present study provided more interesting data. Significant correlations were noted between histology and both P index (negative) and TTP index (positive). The areas under the ROC curves for the P index and TTP index were significant at an established cut-off level that classified Thy 3 nodules as malignant when the P index was lower than 0.99 (sensitivity 37.7%; specificity 75.5%) and the TTP index was greater than 0.98 (sensitivity 56.6%; specificity 75.5%). Our experience of Thy 3 nodules seems to indicate a greater diagnostic role of semi-quantitative USE than of CEUS. In the study by Deng et al. [39], however, quantitative USE and CEUS displayed the same value in distinguishing between benign and malignant nodules.

The simultaneous evaluation of different indicators from US, USE and CEUS and the use of validated cut-off levels for interpreting data – without or with low subjectivity – is crucial in clinical decision-making when cytological results are indeterminate and lobectomy or thyroidectomy is indicated by the guidelines. In the 51 cases that were fully evaluable, our study indicates that the diagnostic power of the cumulative pre-surgical analysis of Thy 3 nodules by means of US, USE and CEUS does not increase sensitivity (64%) but improves specificity (92%); moreover, it showed interesting levels of PPV (75%) and accuracy (84%). While false negative (25%) and false positive (13%) results are limitations, these preliminary data on the combination of US, USE and CEUS with FNAB seem to provide promising indications that unnecessary lobectomy/thyroidectomy can be reduced. To our knowledge there are no studies on Thy 3 nodules in which results obtained from conventional US, USE and CEUS have been simultaneously scored in order to make a clinical decision. In our opinion, US-guided FNAB remains the gold standard in solid nodules. However, when an indeterminate response emerges from cytology, surgery may be postponed if no suspicious findings are observed (in our hands: US score ≤ 2, ELX 2/1 <0.95, P index >0.99 and TTP index <0.98).

There are some limitations to this study. First, the number of fully evaluable Thy 3 nodules was small. As the thyroid tissue adjacent to the Thy 3 nodule may not be normal, both USE and CEUS may sometimes be impracticable. Moreover, injection of the contrast agent before CEUS is sometimes refused by the patient. Second, we did not divide FNAB results into Thy 3 atypia and Thy 3 follicular neoplasm, as suggested by the BTA in 2014 [27,43]. Larger numbers of Thy 3 nodules could necessary in order to compare USE and CEUS data in these two Thy 3 subpopulations. Third, genetic markers were not considered in the present study; however, BRAF, the most frequent mutation in thyroid cancer, seems to be less important in follicular neoplasms [31]. Fourth, this was a single-centre study. However, pooling data from different centres for qualitative and semi-quantitative USE remains impracticable, since different devices may yield various types of strain indices, such as maximum ratio or simple ratio. While we used an average ELX 2/1 index from at least 3 measures, it is unclear whether the average index is truly representative of nodular stiffness. Among our Thy 3 lesions, medullary carcinomas and follicular carcinomas were rare (25% of Thy 3 nodules with histological malignancy). According to some authors [34-36,39], however, false negative results from these tumours should be taken into consideration when USE and CEUS data are evaluated.

In conclusion, in cytologically Thy 3 nodules, conventional US must be associated, when feasible, to the combined application of USE and CEUS. The use of a cut-off based on histology can reduce the surgical approach. Observation should be the first choice when not all instrumental results are suspect.
